# Telocinobufagin inhibits the epithelial-to-mesenchymal transition of breast cancer cells through the PI3K/Akt/Snail signaling pathway

**DOI:** 10.3892/ol.2021.12503

**Published:** 2021-01-31

**Authors:** Yuxue Gao, Lihong Shi, Zhen Cao, Xuetao Zhu, Feng Li, Ruyan Wang, Jinyuan Xu, Jinyi Zhong, Baogang Zhang, Shijun Lu

Oncol Lett 15: 7837-7845, 2018; DOI: 10.3892/ol.2018.8349

Subsequently to the publication of the above paper, an interested reader drew to the authors’ attention that, on p. 7483, the data panel shown for the ‘Fibronectin, 10 μM TBG/mice’ experiment in Fig. 4C appeared to be overlapping with the data panel shown for the ‘p-Erk, Control’ experiment in Fig. 4D, although the coloration of the panels differed slightly.

The authors have re-examined their data, and realized that they copied and pasted the wrong photo for the ‘pErk, control’ panel in Fig. 4D; however, they retained their original data, and the corrected version of the Fig. 4 is shown on the next page.

The authors regret the error that was made in the preparation of the published figure, and confirm that this error did not affect the conclusions reported in the study. The authors are grateful to the editor of *Oncology Letters* for allowing them the opportunity to publish a Corrigendum, and all the authors agree to this Corrigendum. Furthermore, they apologize to the readership for any inconvenience caused.

## Figures and Tables

**Figure 4.**
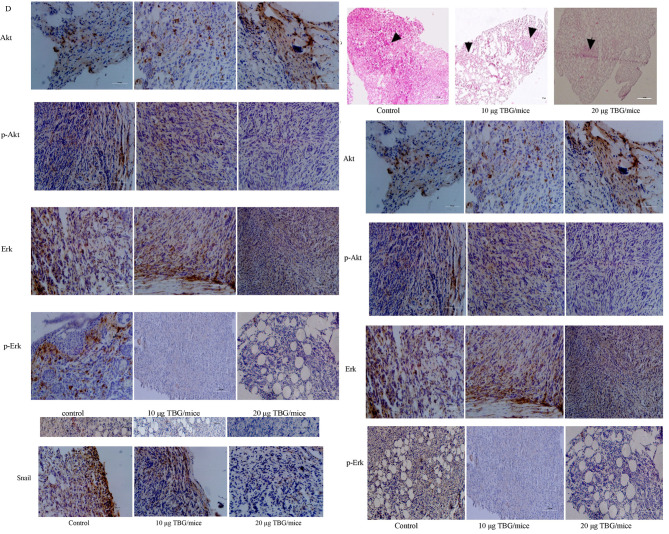
The anti-metastatic effect of TBG in a mouse 4T1 breast tumor model. (A) The body weight of mice was measured 2 times a week and the wet tumor weight was calculated. *P<0.05 vs. control. The results are presented as the mean ± standard error of the mean. (B) Histopathological images of lung sections. Arrows indicate the tumor cells in nodules. (C) Immunohistochemical staining for EMT markers in tumor sections. (D) Immunohistochemical staining for Akt/ERK signaling pathway elements in tumor sections. TBG, telocinobufagin; EMT, epithelial-to-mesenchymal transition; Akt, protein kinase B; ERK, extracellular signal-related kinase.

